# Isolation of a Cyclic (Alkyl)(amino)germylene

**DOI:** 10.3390/molecules21080990

**Published:** 2016-07-29

**Authors:** Liliang Wang, Yi Shan Lim, Yongxin Li, Rakesh Ganguly, Rei Kinjo

**Affiliations:** 1 Division of Chemistry and Biological Chemistry, School of Physical and Mathematical Sciences, Nanyang Technological University, Nanyang Link 21, Singapore 637371, Singapore; lwang018@e.ntu.edu.sg (L.W.); limy0252@e.ntu.edu.sg (Y.S.L.); 2 NTU-CBC Crystallography Facility, Nanyang Technological University Nanyang Link 21, Singapore 637371, Singapore; yongxin@ntu.edu.sg (Y.L.); rganguly@ntu.edu.sg (R.G.)

**Keywords:** germylene, low-valent germanium, sulfur, TEMPO, nitrous oxide

## Abstract

A 1,4-addition of a dichlorogermylene dioxane complex with α,β-unsaturated imine **1** gave a dichlorogermane derivative **2** bearing a GeC_3_N five-membered ring skeleton. By reducing **2** with KC_8_, cyclic (alkyl)(amino)germylene **3** was synthesized and fully characterized. Germylene **3** readily reacted with TEMPO, N_2_O and S_8_, producing the 1:2 adduct **4**, the oxo-bridged dimer **5** and the sulfido-bridged dimer **6**, respectively.

## 1. Introduction

**Figure 1:**
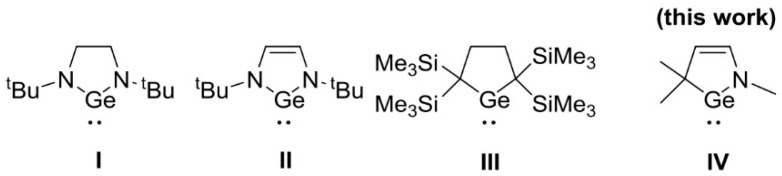
Selected examples of structurally characterized germylenes **I**–**III** featuring five-membered ring skeleton, and this work.

Since the seminal work by Harris and Lappert, who discovered stable organogermylenes in the 1970s [1,2,3], a plethora of studies on the synthesis and reactivity of isolable germylenes have been reported [4,5,6,7,8,9]. Among them, the most extensive investigation has been on *N*-heterocyclic germylenes such as **I**–**II** (Figure 1) that are thermodynamically stabilized by incorporation of two N atoms in a position α to the germylene center [10,11]. In recent years, it has been demonstrated that germylenes can be widely utilized in a variety of applications, ranging from bond activation to polymer chemistry, in addition to their use as ligands for metal complexes [12,13,14,15,16,17,18,19,20,21,22]. Nevertheless, only a few approaches have been reported thus far to expand the diversity of cyclic germylene frameworks [23,24,25,26,27,28,29,30,31,32,33]. In this respect, Kira and co-workers elegantly developed a cyclic dialkylgermylene (**III**, Figure 1) [34]. We envisioned that by replacing only one of the amino substituents of a classical *N*-heterocyclic germylene such as **I**–**II** with an alkyl group, the electronic features and steric impact of the Ge center could be finely modulated. Indeed, in carbene chemistry Bertrand and co-workers have successfully synthesized cyclic (alkyl)(amino)carbenes (CAACs) [35,36,37,38,39,40], and demonstrated that CAACs exhibit both stronger σ-donating and π-accepting characters than classical imidazolin-2-ylidenes, the so-called NHCs. Herein, we reported the preparation, single crystal X-ray diffraction, and computational studies of a cyclic (alkyl)(amino)germylene **IV** (Figure 1).

**Figure 2:**
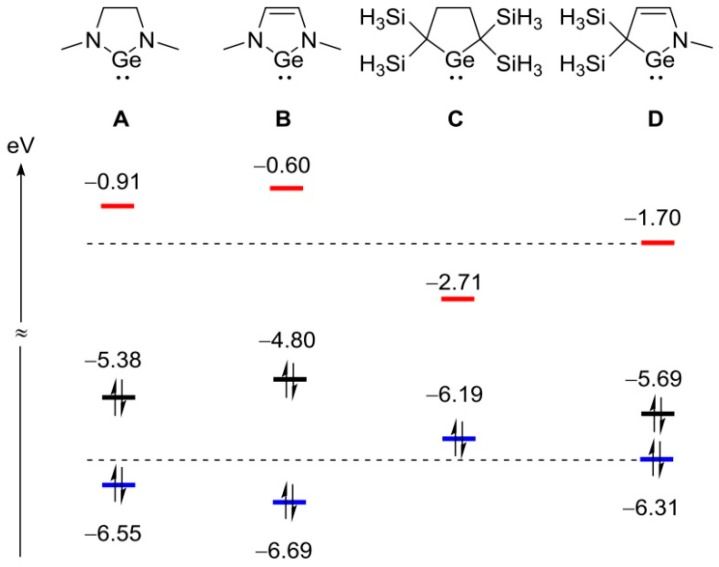
Energy (eV) of frontier orbitals of *N*-heterocyclic germylenes **A**, **B**, cyclic dialkylgermylene **C** and (alkyl)(amino)germylene **D**, calculated at the B3LYP/6-31G(d,p) level of theory.

Brief inspection of the molecular orbitals (MOs) of germylenes **A**–**D** revealed that the HOMO–1 of **A**, **B** and **D** corresponding to the germanium lone pair increases in energy in the order **B** < **A** < **D** (Figure 2). Their HOMOs are mainly centred on the π-system involving the lone pair of the N atoms, and the energies increase in the order **D** < **A** < **B**. In contrast, the Ge lone pair of **C** is found in the HOMO. The energies of the LUMOs, which are predominantly unoccupied p-orbitals on the Ge center for all germylenes **A**–**D**, decrease in the order **B** > **A** > **D** > **C** from −0.60 eV to −2.71 eV. This short analysis indicates that replacement of a π-electron donating and σ-withdrawing amino group by a σ-donating alkyl group (**D**) enhances the electrophilicity of the germylene center, and concomitantly raises the energy of the orbital involving the lone-pair electrons at the germanium center. Note that due to the presence of only one quaternary carbon atom bonding to the Ge atom, the steric profile at the Ge center in **D** differs from those in **A**–**C**.

## 2. Results

**Scheme 1:**
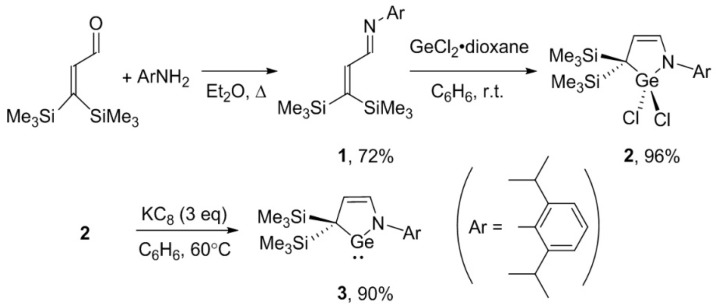
Synthesis of **2** and **3**.

Our strategy for the synthesis of type-**IV** germylene is based on oxidative 1,4-addition of germylene with a 1,3-diene [41,42] and subsequent reduction. We envisaged that dichlorogermylene would undergo cyclization with an α,β-unsaturated imine to afford a dichlorogermane precursor featuring a GeC_3_N five-membered ring skeleton, which could be reduced to access a type-**IV** germylene. To bear out this hypothesis, we carried out the reaction of 3,3-bis(trimethylsilyl)-acrylaldehyde and 2,6-diisopropylaniline in Et_2_O, from which α,β-unsaturated imine **1** was obtained in 72% yield (Scheme 1). Treatment of a stoichiometric amount of dichlorogermylene dioxane complex with **1** in benzene resulted in a 1,4-addition via a redox reaction between them, which gave the cyclic dichlorogermane derivative **2** in 96% yield. The result represents the first example of a 1,4-addition of Cl_2_Ge: to an α,β-unsaturated imine. A mixture of **2** and three equivalents of potassium graphite was heated at 60 °C in benzene. After work-up, germylene **3** was isolated as a dark red solid in 90% yield. In the ^1^H-NMR spectrum of **3**, a Me_3_Si group singlet appears at 0.21 ppm, which is identical to that 0.22 ppm seen for **2**. The ^13^C-NMR spectrum of **3** displays two peaks at 118.9 and 146.7 ppm for the C(sp^2^)-H carbon atoms in the GeNC_3_ five-membered ring, which are shifted significantly downfield in comparison to those of **2** (99.0 ppm and 139.1 ppm).

**Figure 3:**
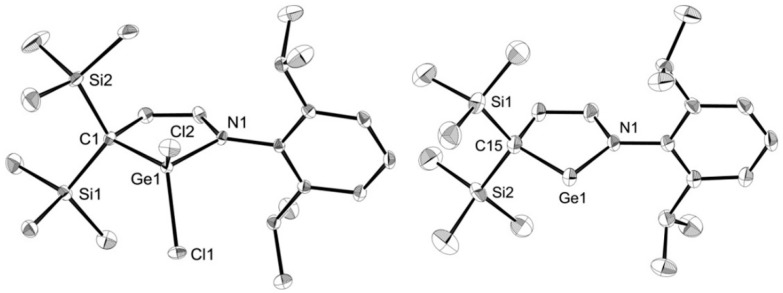
Solid state structures of **2** (**left**) and **3** (**right**). Hydrogen atoms are omitted for clarity. Thermal ellipsoids are shown at the 50% probability level.

The solid state molecular structures of compounds **2** and **3** were determined by single crystal X-ray diffraction analysis (Figure 3). Five atoms in the GeNC_3_ five-membered ring of **3** are coplanar (the sum of internal pentagon angles = 539.91°), and 2,6-diisopropylphenyl ring at the N1 atom and the GeNC_3_ skeleton are nearly perpendicular to each other with the twist angle of 89.86°. The Ge1-N1 bond (1.859(11) Å) and the Ge1-C15 bond (1.998(14) Å) distances are slightly longer than those [Ge1-N1 1.822(2) Å, Ge1-C1 1.959(3) Å] of **2**. The N1-Ge1-C15 bond angle of 86.5(5)° in **3** is more acute than the N1-Ge1-C1 bond angle [94.90(11)°] in **2**, the corresponding N-Ge-N bond angle [84.8(1)°] in **II** [10] as well as the corresponding C-Ge-C bond angle [90.97(9)°] in **III** [34]. 

**Figure 4:**
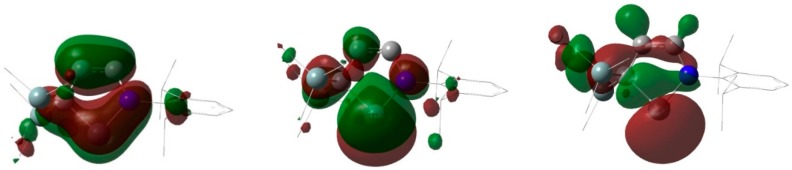
Plots of the HOMO (**left**), LUMO (**middle**) and HOMO–1 (**right**) of **3** calculated at the B3LYP/6-31G(d,p) level of theory (hydrogen atoms are omitted for clarify).

To gain insight into the electronic features, we carried out quantum chemical calculations on 3. A lone-pair orbital at the Ge atom was found in the HOMO–1, whereas the HOMO displays a Ge-N π-bonding involving the two C-Si σ-bonds which exhibits anti-bonding conjugation with the π-orbital of the C=C moiety (Figure 4).

**Scheme 2:**
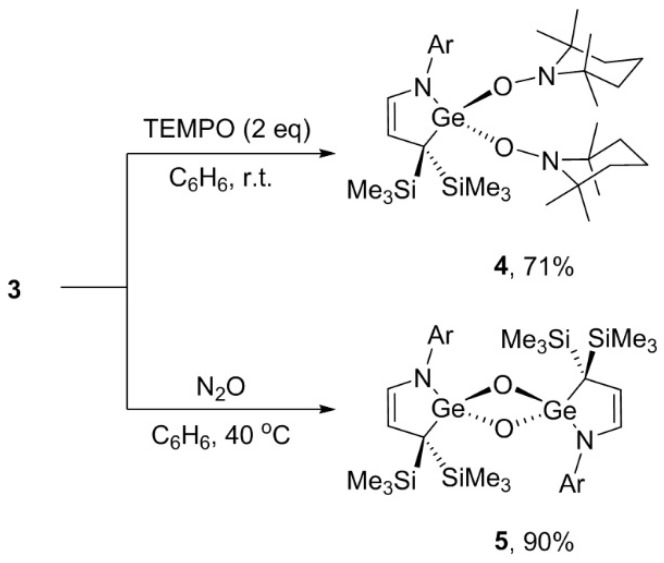
Reactions of **3** with TEMPO, N_2_O.

**Figure 5:**
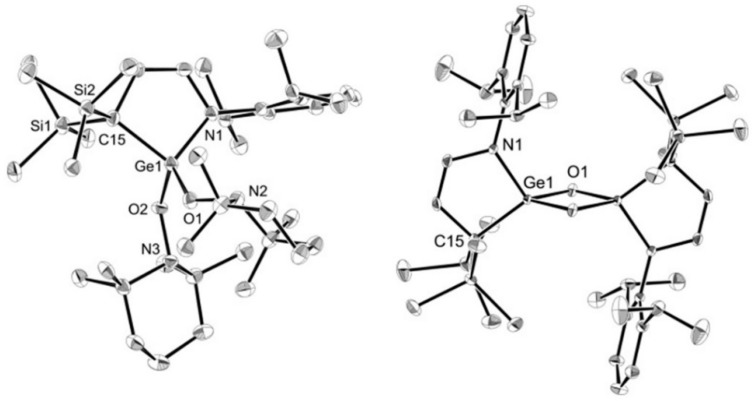
Solid state structures of **4** (**left**) and **5** (**right**). Hydrogen atoms are omitted for clarity. Thermal ellipsoids are shown at the 50% probability level.

The LUMO is mainly the empty p orbital of the Ge center. Natural bond orbital (NBO) analysis gave Wiberg bond index (WBI) values of the Ge-N bond (0.78) and the Ge-C bond (0.73), indicating single bond nature of these bonds. Natural population analysis (NPA) manifested the second order perturbation energy of 33.97 kcal·mol^−1^ for the interaction between a lone-pair on the N atom and formally unoccupied p-orbital of the Ge atom. A UV-vis spectrum of **3** in hexane exhibits a broad peak with λ_max_ at 368 nm, which is due to the HOMO-LUMO transition, responsible for the deep red colour of **3**. Compound **3** is stable at room temperature, both in the solid state and in solutions (benzene and THF), but it rapidly decomposes upon exposure to air, indicating its high reactivity. Then, we turned our interest into the reactivity of **3**. We inferred that the Ge(II) center in **3** would undergo redox reactions with appropriate oxidants [43,44,45]. Indeed, by analogy to dialkylgermylene **III** [46,47], compound **3** readily reacted with two equivalents of 2,2,6,6-tetramethylpiperidine N-oxide (TEMPO) under ambient conditions, to afford **4** in 71% yield (Scheme 2). We also examined the 1:1 reaction between **3** and TEMPO, which again gave **4**, and unreacted **3** was recovered after the reaction. Kira and co-workers reported that the thermal reaction of the 1:2 **III**·(TEMPO)_2_ adduct yielded the corresponding 1,3-digemadioxetane [**III**-O]_2_ [46,47]. By contrast, thermolysis of **4** in C_6_D_6_ led to the formation of a complex mixture. Meanwhile, reaction of **3** and nitrous oxide (N_2_O) at 40 °C proceeded smoothly, and the corresponding 1,3-digemadioxetane **5** was obtained as a white solid in 90% yield [48,49,50,51,52]. Notably, Driess et al. reported that six-membered ring *N*-heterocyclic germylene is resistant towards N_2_O [53,54,55,56], which is in marked contrast to our result. Smooth transfer of an O atom from N_2_O to **3** is probably due to the enhanced Lewis acidic nature of the Ge center attributed to the low-lying LUMO, which is in line with our DFT calculation results (Figure 1). Compounds **4** and **5** were fully characterized by standard spectroscopic methods and X-ray diffraction analysis (Figure 5). The Ge-O bond distances (1.814(3) Å and 1.796(3) Å) in **4** are lengthened with respect to the Ge=O double bond distance of 1.6468(5) Å in (Eind)_2_Ge=O (Eind = 1,1,3,3,5,5,7,7-octaethyl-*s*-hydrindacen-4-yl group) [57], and comparable to those (1.824(2) Å and 1.826(2) Å) in **III**(TEMPO)_2_ [46,47]. Both the Ge-C bond (2.020(4) Å) and Ge-N bond (1.871(3) Å) distances are similar to those of the corresponding bonds in **3**. Compound **5** displays a spiro-structure involving a nearly planar Ge_2_O_2_ four-membered ring in which two 2,6-diisopropylphenyl groups are located on the opposite side with respect to the Ge_2_O_2_ plane. The O-Ge-O and Ge-O-Ge bond angles are 86.35(8)° and 93.65(8)°, respectively. The Ge-O bond distances (1.8084(16) Å and 1.8090(17) Å) are almost identical to those (1.821(2) Å and 1.822(2) Å) in [**III**-O]_2_ [46,47]. 

**Scheme 3:**
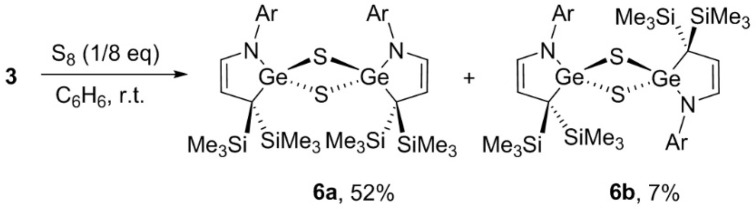
Reactions of **3** with S_8_.

Successful transfer of oxygen atom from N_2_O to **3** prompted us to further investigate the reaction with elemental sulfur. A mixture of **3** and S_8_ (1/8 eq) in benzene was stirred at ambient temperature for 1 h. After work-up, sulfido-bridged dimers **6** were obtained as a mixture of two diastereomers in 59% total yield (Scheme 3) [58,59,60]. Facile oxidation of the Ge center by S_8_ supports the electron-donating ability of **3**.

**Figure 6:**
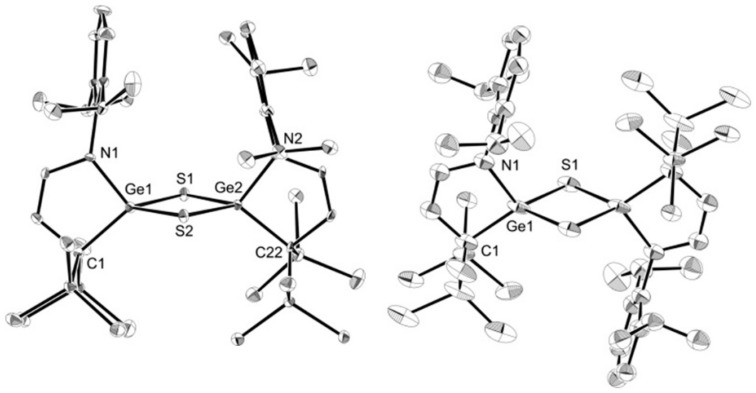
Solid state structures of **6a** (**left**) and **6b** (**right**). Hydrogen atoms are omitted for clarity. Thermal ellipsoids are shown at the 50% probability level.

Interestingly, purification of **6a** and **6b** by column chromatography and subsequent X-ray crystallographic analysis (Figure 6) confirmed that the major product is **6a** bearing two 2,6-diisopropylphenyl groups located on the same side of the Ge_2_S_2_ plane, which is in sharp contrast to the stereoselective formation of **5** having a similar geometry to **6b**. Both **6a** and **6b** involve a planar Ge_2_S_2_ four-membered ring. The S-Ge-S and Ge-S-Ge bond angles are 93.47(5)° and 86.56(5)° for **6a**, and 94.01(12)° and 85.99(12)° for **6b**, respectively. The Ge-S bond distances (**6a**: 2.2462(13) Å and 2.2406(13) Å, **6b**: 2.231(3) Å and 2.237(3) Å) are in the range reported of Ge-S single bonds (2.17–2.25 Å) [61], and longer than the Ge=S double bond length of 2.049(3) Å in [Tbt(Trip)Ge=S] (Tbt = 2,4,6-{(Me_3_Si)_2_CH}_3_C_6_H_2_, Trip = 2,4,6-^i^Pr_3_C_6_H_2_) [62]. To investigate the reaction mechanism, we attempted to quench the reaction intermediate employing Lewis bases (pyridine, Me_3_N, Ph_3_P and Et_3_N) and Lewis acids (BPh_3_ and B(C_6_F_5_)_3_) [63,64,65]. However, none of them afforded any trapped products, and only the formation of **6a** and **6b** were observed.

## 3. Materials and Methods

### 3.1. General Information

All reactions were performed under an atmosphere of argon by using standard Schlenk or dry box techniques. Solvents were dried over Na metal, K metal or CaH_2_. All the substrates were obtained from commercial sources or synthesized following literature procedures. Analytical thin layer chromatography was performed on trimethylamine-deactivated 0.25 mm silica gel 60-F254 plates (Merck, Darmstadt, Germany). Visualization was carried out with UV light. ^1^H (400 MHz), and ^13^C (100 MHz) spectra were obtained with an AVIII 400 MHz BBFO1 spectrometer (Bruker Instruments, Karlsruhe, Germany) at 298 K. NMR multiplicities are abbreviated as follows: s = singlet, d = doublet, t = triplet, m = multiplet, br = broad signal. Coupling constants *J* are given in Hz. Electrospray ionization (ESI) mass spectra were obtained at the Mass Spectrometry Laboratory at the Division of Chemistry and Biological Chemistry, Nanyang Technological University. Melting points were measured with an OpticMelt system (Stanford Research Systems, Sunnyvale, CA, USA).

^1^H-, and ^13^C-NMR spectra of all compounds are provided in Supplementary Material (Figures S1–S16).

### 3.2. Synthesis of ***1***

2,6-Diisopropylaniline (55 mmol, 9.75 g) and *3*,*3*-bis(trimethylsilyl)acrylaldehyde [66] (50 mmol, 10.00 g) were mixed in ether (100 mL). After the mixture was refluxed overnight, the product was purified by silica gel column chromatography (petroleum ether/ethyl acetate= 10:1) to afford **1** as a yellow solid (12.9 g, 72%). Mp: 109 °C; ^1^H-NMR (CDCl_3_) δ 8.13 (d, *J* = 10.1 Hz, 1H, C*H*), 7.36 (d, *J* = 10.1 Hz, 1H, C*H*), 7.18–7.07 (m, 3H, Ar-*H*), 2.91 (dt, *J* = 13.7, 6.9 Hz, 2H, C*H*), 1.17 (s, 6H, C*H*_3_), 1.15 (s, 6H, C*H*_3_), 0.24 (s, 9H, Si(C*H*_3_)_3_), 0.21 (s, 9H, Si(C*H*_3_)_3_); ^13^C-NMR (CDCl_3_) δ 163.40 (*C*H), 162.04 (*C*^q^), 151.12 (*C*H), 148.79 (*C*^q^), 137.23 (*C*^q^), 124.26 (*C*^q^), 122.96 (*C*H), 27.73 (*C*H), 23.61 (*C*H_3_), 2.39 (Si(*C*H_3_)_3_), 0.05 (Si(*C*H_3_)_3_); HRMS (ESI): *m/z* calcd for C_21_H_38_NSi_2_: 359.2465 [(M + H)]^+^; found: 359.2468.

### 3.3. Synthesis of ***2***

Compound **1** (4 mmol, 1.42 g) and dichlorogermadiyl dioxane (4 mmol, 0.93 g) were mixed in benzene (20 mL). The mixture was stirred at room temperature overnight, and then all volatiles were removed under vacuum to afford **2** as a yellow solid (1.93 g, 96%). Mp: 154 °C; ^1^H-NMR (C_6_D_6_) δ 7.15–6.88 (m, 3H, Ar-*H*), 6.30 (d, *J* = 6.3 Hz, 1H, C*H*), 4.60 (d, *J* = 6.3 Hz, 1H, C*H*), 3.48 (dt, *J* = 13.7, 6.9 Hz, 2H, C*H*), 1.31 (d, *J* = 6.9 Hz, 6H, C*H*_3_), 1.14 (d, *J* = 6.9 Hz, 6H, C*H*_3_), 0.22 (s, 18H, Si(C*H*_3_)_3_); ^13^C-NMR (C_6_D_6_) δ 147.82 (*C*^q^), 139.06 (*C*H), 137.11 (*C*^q^), 127.91 (*C*H), 124.04 (*C*H), 98.98 (*C*H), 33.96, (*C*^q^), 28.04 (*C*H), 25.72 (*C*H_3_), 23.06 (*C*H_3_), 0.47 (Si(*C*H_3_)_3_); HRMS (ESI): *m/z* calcd for C_21_H_38_NSi_2_Cl_2_Ge: 504.1132 [(M + H)]^+^; found: 504.1156.

### 3.4. Synthesis of ***3***

Compound **2** (2 mmol, 1.02 g) and KC_8_ (6 mmol, 0.81 g) were mixed in benzene (30 mL). The mixture was stirred at 60 °C for 6 h. After stirring for another 30 min at room temperature, the mixture was filtered and then the solution was concentrated under vacuum which afforded **3** as a dark red solid (0.78 g, 90%). Mp: 95 °C; ^1^H-NMR (C_6_D_6_) δ 7.22 (d, *J* = 4.1 Hz, 1H, C*H*), 7.22–7.14 (m, 3H, Ar-*H*), 6.08 (d, *J* = 4.1 Hz, 1H, C*H*), 3.25 (dt, *J* = 13.8, 6.9 Hz, 2H, C*H*), 1.21 (d, *J* = 6.9 Hz, 6H, C*H*_3_), 1.17 (d, *J* = 6.9 Hz, 6H, C*H*_3_), 0.21 (s, 18H, Si(C*H*_3_)_3_); ^13^C-NMR (C_6_D_6_) δ 146.70 (*C*H), 144.51 (*C*^q^), 143.89 (*C*^q^), 126.87 (*C*H), 123.30 (*C*H), 118.94 (*C*H), 27.94 (*C*H), 24.94 (*C*H_3_), 23.83 (*C*H_3_), 1.78 (Si(*C*H_3_)_3_); HRMS (ESI): *m/z* calcd for C_21_H_38_NSi_2_Ge: 434.1755 [(M + H)]^+^; found: 434.1761.

### 3.5. Reaction of ***3*** with TEMPO

TEMPO (0.50 mmol, 78 mg) and **3** (0.23 mmol, 100 mg) were mixed in benzene (2 mL) and stirred for 5 min at room temperature. After the solvent was removed under vacuum, the residue was recrystallized from hexane to afford **4** as colorless crystals (120 mg, 71%). Mp: 195 °C. ^1^H-NMR (C_6_D_6_) δ 7.18 (s, 3H, Ar-*H*), 6.43 (d, *J* = 6.6 Hz, 1H, C*H*), 4.66 (d, *J* = 6.6 Hz, 1H, C*H*), 3.87 (s, 2H, C*H*), 2.15–0.87 (m, 48H), 0.47 (s, 18H, Si(C*H*_3_)_3_); ^13^C-NMR (C_6_D_6_) δ 147.32 (*C*^q^), 144.21 (*C*^q^), 140.67 (*C*H), 126.43 (*C*H), 123.63 (*C*H), 97.13 (*C*H), 61.35 (*C*^q^), 61.15 (*C*^q^), 41.85 (*C*H_3_), 41.35 (*C*H_3_), 34.30 (*C*H_2_), 28.10 (*C*H_2_), 26.47 (*C*H), 21.83 (*C*H_2_), 16.96 (*C*H_3_), 4.16 (Si(*C*H_3_)_3_); HRMS (ESI): *m/z* calcd for C_39_H_74_N_3_O_2_Si_2_Ge: 746.4531. [(M + H)]^+^; found: 746.4509.

### 3.6. Reaction of ***3*** with N_2_O

Nitrous oxide (0.23 mmol, 10 mg) was condensed at liquid nitrogen in a benzene solution of **3** (0.23 mmol, 100 mg). The reaction mixture was heated at 40 °C and stirred for 1 h. After the solvent was removed under vacuum, recrystallization of the residue from benzene afforded **5** as colorless crystals (93 mg, 90%). Mp: 278 °C (dec). ^1^H-NMR (C_6_D_6_) δ 7.19 (m, 2H, Ar-H), 7.15–7.11 (m, 4H, Ar-H), 6.34 (d, *J* = 6.3 Hz, 2H, CH), 4.65 (d, *J* = 6.3 Hz, 2H, CH), 3.50 (dt, *J* = 13.6, 6.8 Hz, 4H, CH), 1.42 (d, *J* = 6.8 Hz, 12H, CH_3_), 1.21 (d, *J* = 6.8 Hz, 12H, CH_3_), 0.05 (s, 36H, Si(CH_3_)_3_); ^13^C-NMR (C_6_D_6_) δ 147.81 (C^q^), 141.12 (CH), 140.44 (C^q^), 123.91 (CH), 120.00 (CH), 97.48 (CH), 28.23 (CH), 26.01 (CH_3_), 22.70 (CH_3_), 0.25 (Si(CH_3_)_3_); HRMS (ESI): *m/z* calcd for C_42_H_75_N_2_O_2_Si_4_Ge_2_: 899.3329. [(M+H)]^+^; found: 899.3361.

### 3.7. Reaction of ***3*** with S_8_

Sulfur (0.03 mmol, 7.4 mg) and **3** (0.23 mmol, 100 mg) were mixed in benzene (2 mL) and the mixture was stirred at room temperature for 1 h. After the solvent was removed under vacuum, the product was purified by silica gel column chromatography (petroleum ether/ethyl acetate = 10:1) to afford colorless crystals of **6a** (56 mg, 52%) and **6b** (7.5 mg, 7%).

**6a**: Mp: 230 °C (dec); ^1^H-NMR (CDCl_3_) δ 7.10 (t, *J* = 7.7 Hz, 2H, Ar-*H*), 6.89 (d, *J* = 7.7 Hz, 4H, Ar-*H*), 6.19 (d, *J* = 6.1 Hz, 2H, C*H*), 4.75 (d, *J* = 6.1 Hz, 2H, C*H*), 3.00 (dt, *J* = 13.6, 6.8 Hz, 4H, C*H*), 0.96 (d, *J* = 6.9 Hz, 12H, C*H*_3_), 0.93 (d, *J* = 6.7 Hz, 12H, C*H*_3_), 0.27 (s, 36H, Si(C*H*_3_)_3_); ^13^C-NMR (CDCl_3_) δ 146.96 (*C*^q^), 140.92 (*C*H), 139.61 (*C*^q^), 126.38 (*C*H), 123.58 (*C*H), 98.98 (*C*H), 28.18 (*C*H), 26.00 (*C*H_3_), 22.91 (*C*H_3_), 1.05 (Si(*C*H_3_)_3_); HRMS (ESI): *m/z* calcd for C_42_H_75_N_2_S_2_Si_4_Ge_2_: 931.2872. [(M + H)]^+^; found: 931.2892.

**6b**: Mp: 237 °C (dec); ^1^H-NMR (CDCl_3_) δ 7.18 (m, 6H, Ar-*H*), 6.32 (d, *J* = 6.0 Hz, 2H, C*H*), 4.68 (d, *J* = 6.0 Hz, 2H, C*H*), 3.24 (dt, *J* = 13.5, 6.8 Hz, 4H, C*H*), 1.32 (d, *J* = 6.8 Hz, 12H, C*H*_3_), 1.04 (d, *J* = 6.8 Hz, 12H, C*H*_3_), −0.01 (s, 36H, Si(C*H*_3_)_3_); ^13^C-NMR (CDCl_3_) δ 147.88 (*C*^q^), 140.31 (*C*H), 139.73 (*C*^q^), 126.90 (*C*H), 123.67 (*C*H), 98.65 (*C*H), 28.55 (*C*H), 26.56 (*C*H_3_), 22.27 (*C*H_3_), 0.61 (Si(*C*H_3_)_3_); HRMS (ESI): *m/z* calcd for C_42_H_75_N_2_S_2_Si_4_Ge_2_: 931.2872. [(M + H)]^+^; found: 931.2898.

### 3.8. X-ray Crystallography

X-ray data collection and structural refinement. Intensity data for compounds **1**–**5**, and **6a**,**b** was collected using a Bruker APEX II diffractometer. The crystals were measured at 103(2) K. The structure was solved by direct phase determination (SHELX-2013) and refined for all data by full-matrix least-squares methods on F2 [67,68]. All non-hydrogen atoms were subjected to anisotropic refinement. The hydrogen atoms were generated geometrically and allowed to ride in their respective parent atoms; they were assigned appropriate isotropic thermal parameters and included in the structure-factor calculations. CCDC; 1447574, 1447575, 1447576, 1447577, 1447578, 1447579 and 1491124 contains the supplementary crystallographic data for this paper. The data can be obtained free of charge from the Cambridge Crystallography Data Center via www.ccdc.cam.ac.uk/data_request/cif.

### 3.9. Theoretical Study

Gaussian 09 was used for all density functional theory (DFT) calculations [69]. Geometry optimization and frequency calculations for **A**, **B**, **C** and **D** were performed at the B3LYP/6-31G(d,p) level of theory. Geometry optimization, frequency calculations, natural bond orbital (NBO) analysis of compound **3** were performed at the B3LYP/6-31G(d,p) level of theory.

## 4. Conclusions

In conclusion, we have developed a direct approach for the synthesis of a dichlorogermanium derivative **2** featuring a five-membered GeNC_3_ ring through simple 1,4-addition of dichloro-germylene dioxane complex with α,β-unsaturated imine **1**. Subsequent reduction of **2** with KC_8_ gave cyclic (alkyl)(amino)germylene (CAAGe) **3** possessing the low-lying LUMO, as supported by DFT calculations. Compound **3** readily reacted with TEMPO and N_2_O. The former afforded 1:2 adduct **4** whereas the latter yielded the oxo-bridged dimer **5** stereoselectively. In contrast, oxidation of **3** with S_8_ led to the formation of the sulfido-bridged dimers **6** as a mixture of two diastereomers involving Ge_2_S_2_ four-membered ring framework. Development of the silicon analogue of compound **3** is currently under investigation in our laboratory.

## Supplementary Materials

Supplementary materials can be accessed at: http://www.mdpi.com/1420-3049/21/8/990/s1.
