# Supplementary material for: Isolation of a Cyclic (Alkyl)(amino)germylene
Source: Molecules. 2016 Jul 29;21(8):990. doi: 10.3390/molecules21080990 (PMC6273211; doi:10.3390/molecules21080990)
Supplement: Supplementary file 1 [file molecules-21-00990-s001.pdf]

Supplementary Materials: Isolation of a Cyclic (Alkyl)(Amino)Germylene

Liliang Wang, Yi Shan Lim, Yongxin Li, Rakesh Ganguly and Rei Kinjo

1. Crystallographic Details

Table S1. X-ray data for **1–5**, and **6a**,**b**.

**Compounds**	**1**	**2**	**3**	**4**			
Formula	$C_{21}H_{37}NSi_2$	$C_{21}H_{37}Cl_2GeNSi_2$	$C_{21}H_{37}GeNSi_2$	$C_{39}H_{73}GeN_3O_2Si_2$	$C_{21}H_{37}GeNOSi_2$	$C_{42}H_{75}Ge_2N_2S_2Si_4$	$C_{42}H_{74}Ge_2N_2S_2Si_4$
Fw	359.69	503.18	432.28	744.77	448.28	929.70	928.69
Crystsyst	monoclinic	monoclinic	orthorhombic	triclinic	triclinic	monoclinic	triclinic
Space group	P 1 21/c 1	P 1 21/n 1	P n m a	P -1	P -1	P 1 21/n 1	P -1
Size (mm³) space group	0.180 × 0.220 × 0.360	0.260 × 0.300 × 0.320	0.220 × 0.300 × 0.420	0.060 × 0.200 × 0.400	0.380 × 0.400 × 0.420	0.220 × 0.300 × 0.420	0.080 × 0.280 × 0.380
T/K	103(2)	103(2)	103(2)	103(2)	103(2)	103(2)	103(2)
a, Å	12.2230(12)	17.7322(15)	18.4853(11)	12.027(3)	10.1766(6)	10.2311(6)	10.505(9)
b, Å	12.3881(14)	7.2799(6)	10.4296(6)	17.639(4)	12.0888(7)	26.3317(18)	11.529(11)
c, Å	15.7504(16)	20.5722(17)	12.7509(8)	19.606(4)	21.8730(14)	18.5201(13)	12.465(11)
α, deg	90	90	90	98.477(2)	80.3144(18)	90	105.094(10)
β, deg	102.165(3)	104.674(3)	90	92.2855(19)	80.8124(18)	91.640(2)	106.866(9)
γ, deg	90	90	90	90.304(2)	65.2397(18)	90	111.153(9)
V, A³	2331.4(4)	2569.0(4)	2458.3(3)	4110.3(15)	2396.7(3)	4987.3(6)	1229.5(19)
Z	4	4	4	4	4	4	1
d_{calcd}g·cm⁻³	1.025	1.301	1.168	1.204	1.242	1.238	1.254
μ, mm⁻¹	0.155	1.501	1.348	0.839	1.388	1.414	1.434
Refl collected	31,602	33,105	18,278	19,943	96,838	75,028	12,834
T_{max}/T_{min}	0.9730/0.9460	0.6960/0.6450	0.7560/0.6010	0.9510/0.7300	0.6210/0.5930	0.7460/0.5880	0.8940/06120
N_{measd}	6254	8173	2656	19943	21,165	15,294	4412
[R int]	0.0749	0.0690	0.1358	0.1425	0.1588	0.1900	0.1661
R [I > 2sigma(I)]	0.0448	0.0521	0.0629	0.0719	0.0606	0.0729	0.0932
R_w [I > 2sigma(I)]	0.1044	0.1085	0.1149	0.1366	0.1240	0.1484	0.2151
GOF	1.033	1.154	1.030	0.919	0.998	0.991	1.020
Largest diff. peak/hole [e·Å⁻³]	0.362/−0.236	1.283/−0.729	0.295/−0.575	0.726/−1.116	0.819/−0.896	2.128/−1.539	1.433/−0.730

2. ¹H- and ¹³C-NMR Spectra

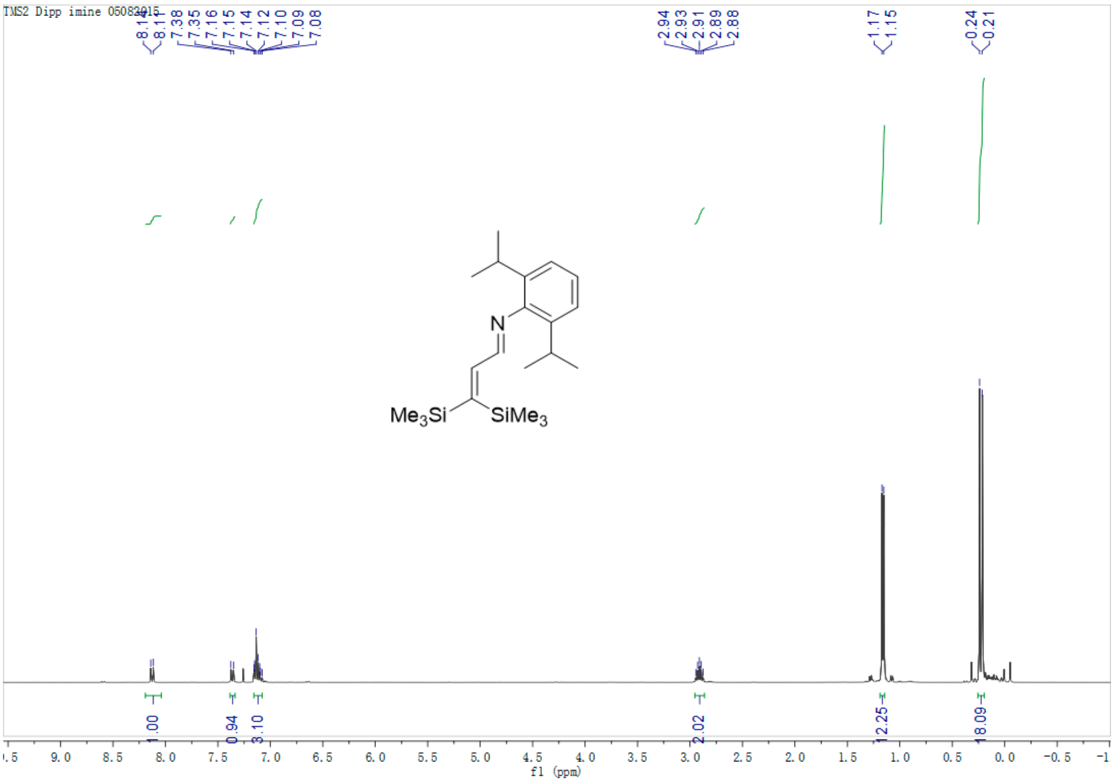

Figure S1. ¹H-NMR spectrum of **1.**

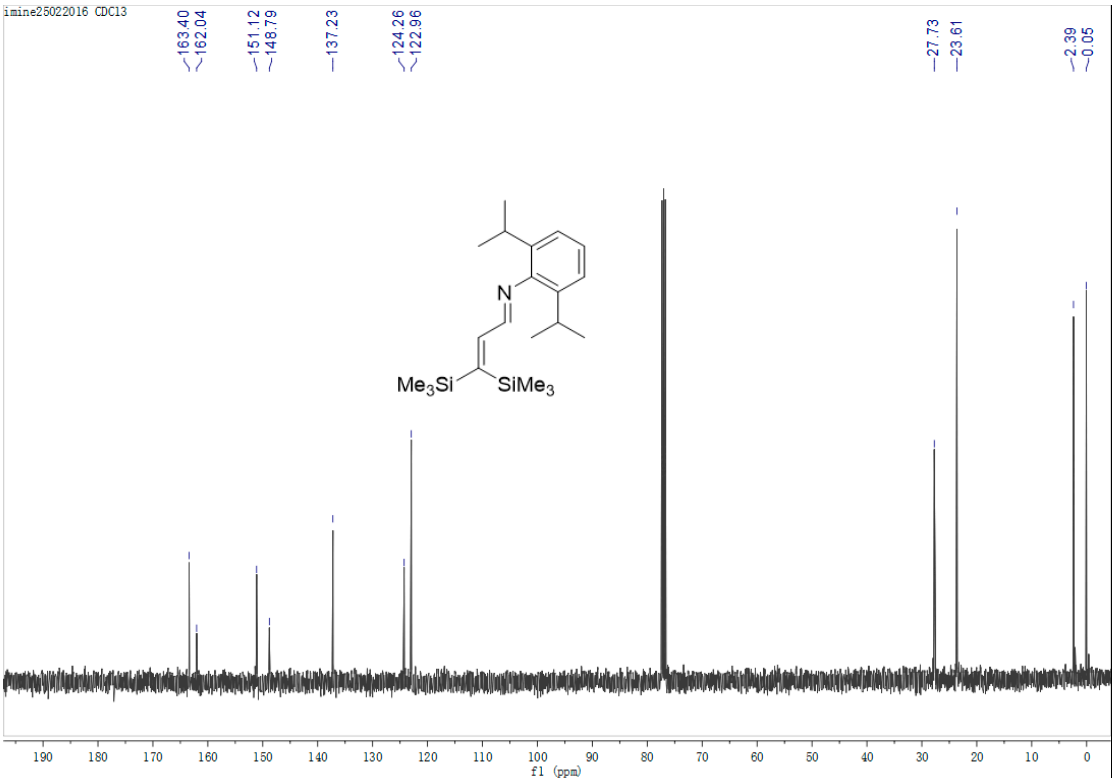

Figure S2. ¹³C-NMR spectrum of **1.**

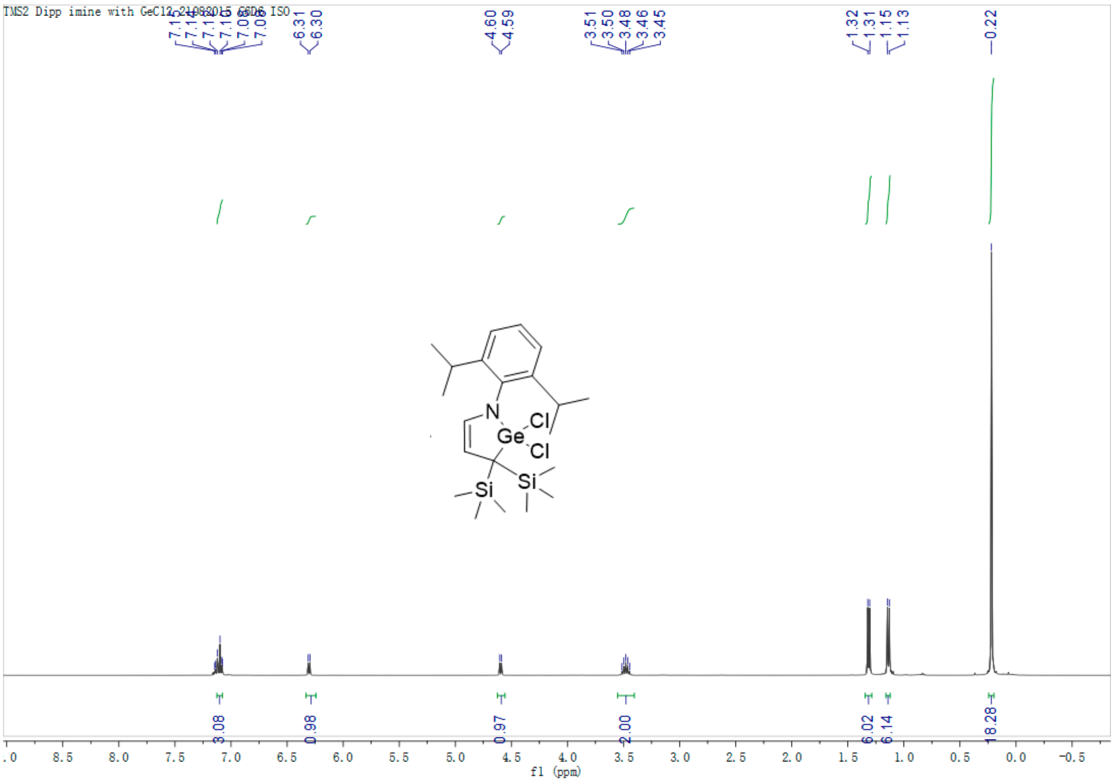

Figure S3. ^{1}H-NMR spectrum of **2.**

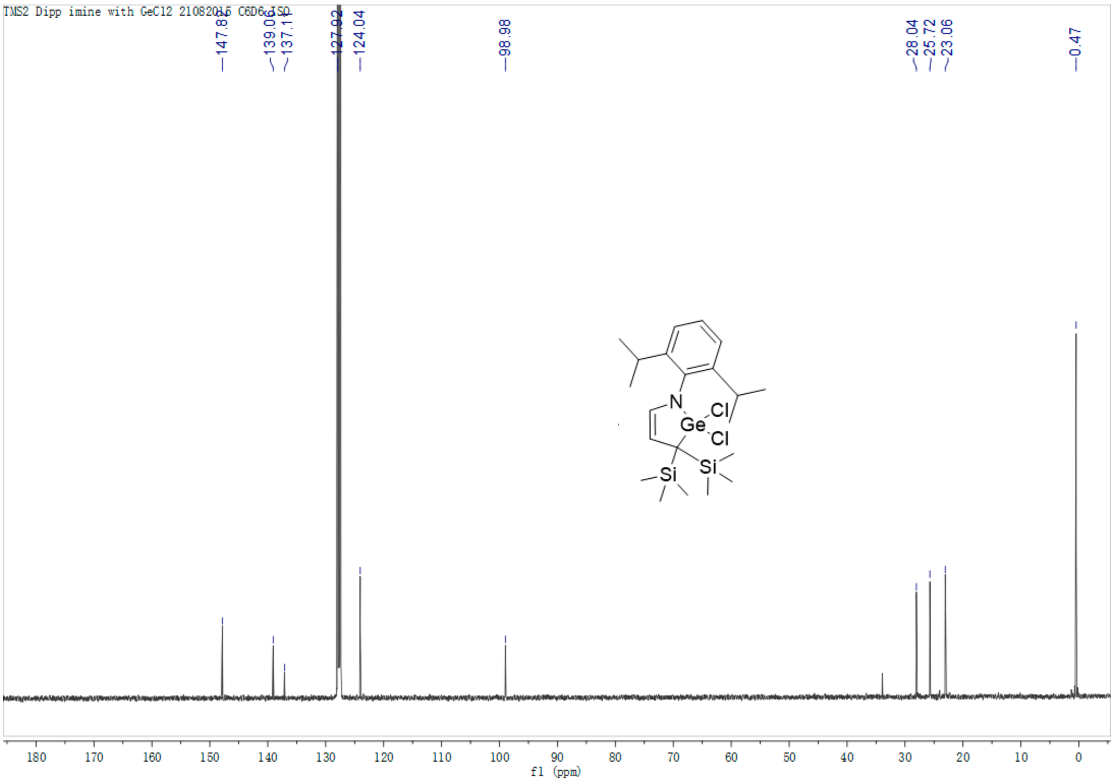

Figure S4. ^{13}C-NMR spectrum of **2.**

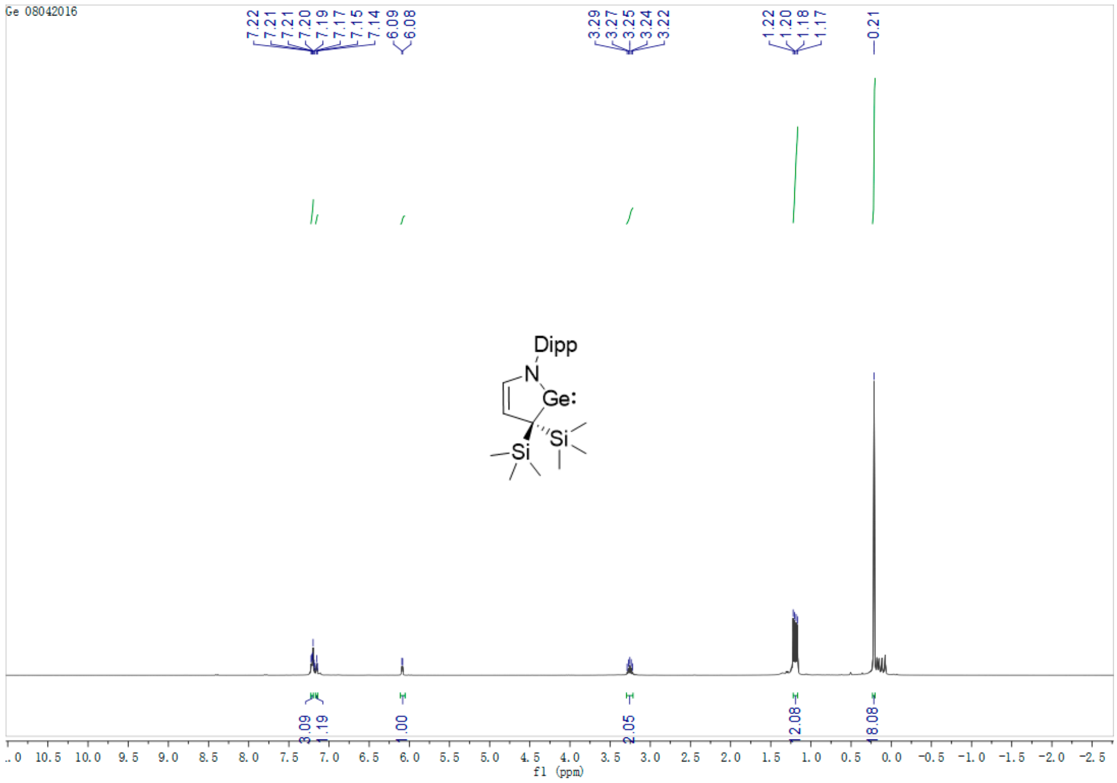

Figure S5. ^{1}H-NMR spectrum of **3.**

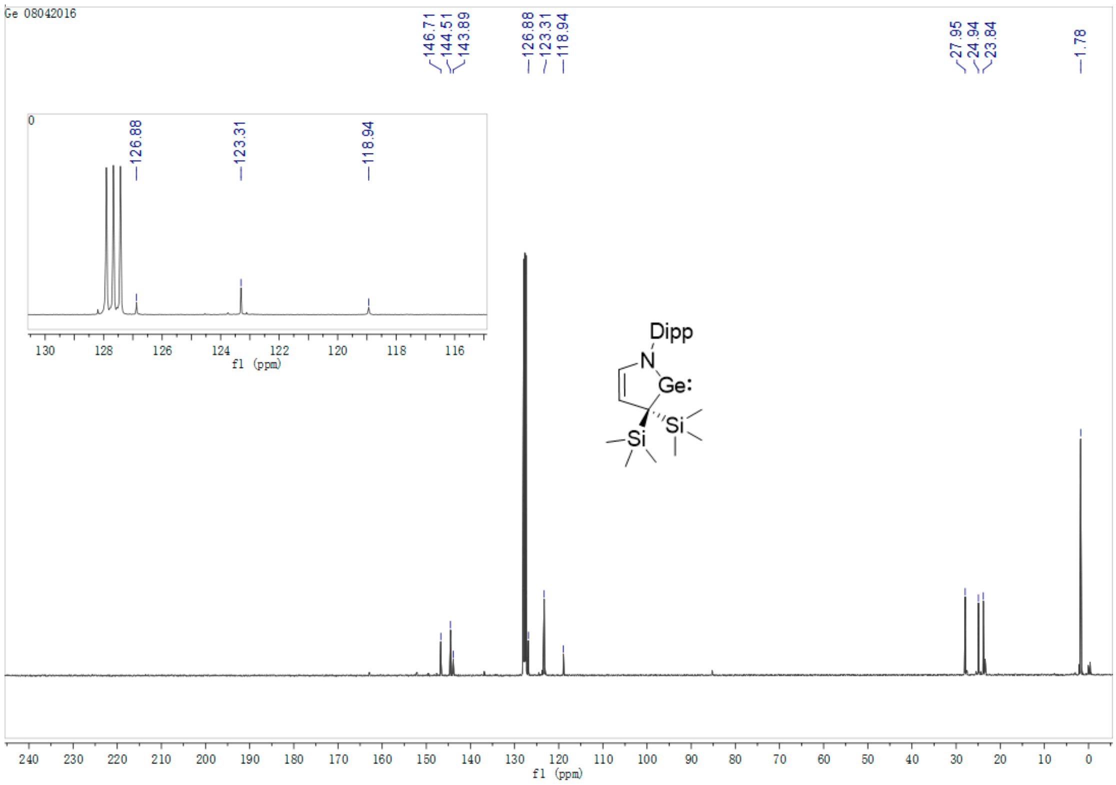

Figure S6. ^{13}C-NMR spectrum of **3.**

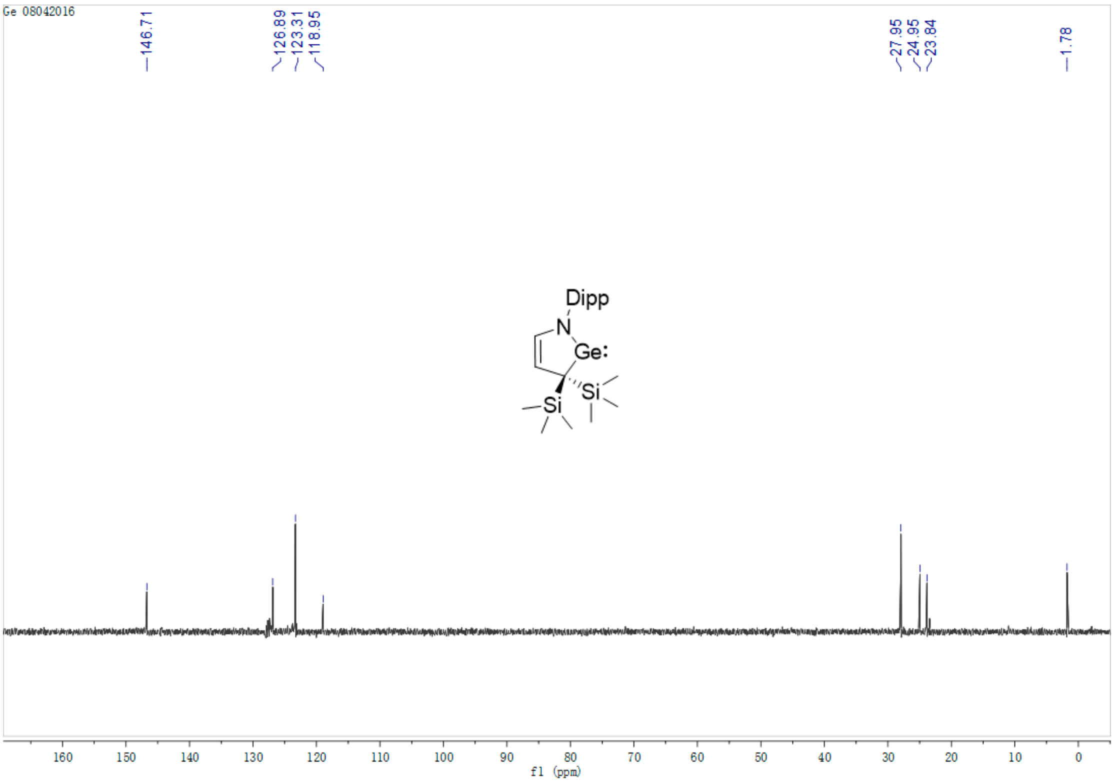

Figure S7. DEPT-135 ^{13}C-NMR spectrum of **3.**

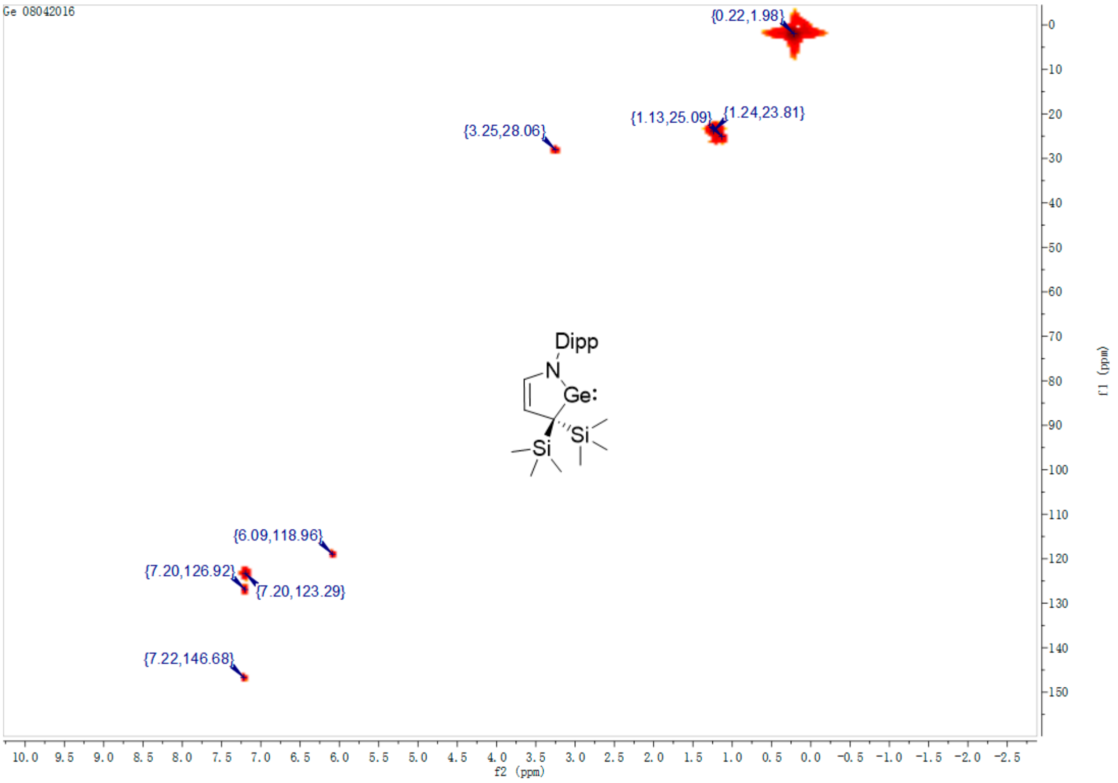

Figure S8. ^{1}H-^{13}C HSQC spectrum of **3.**

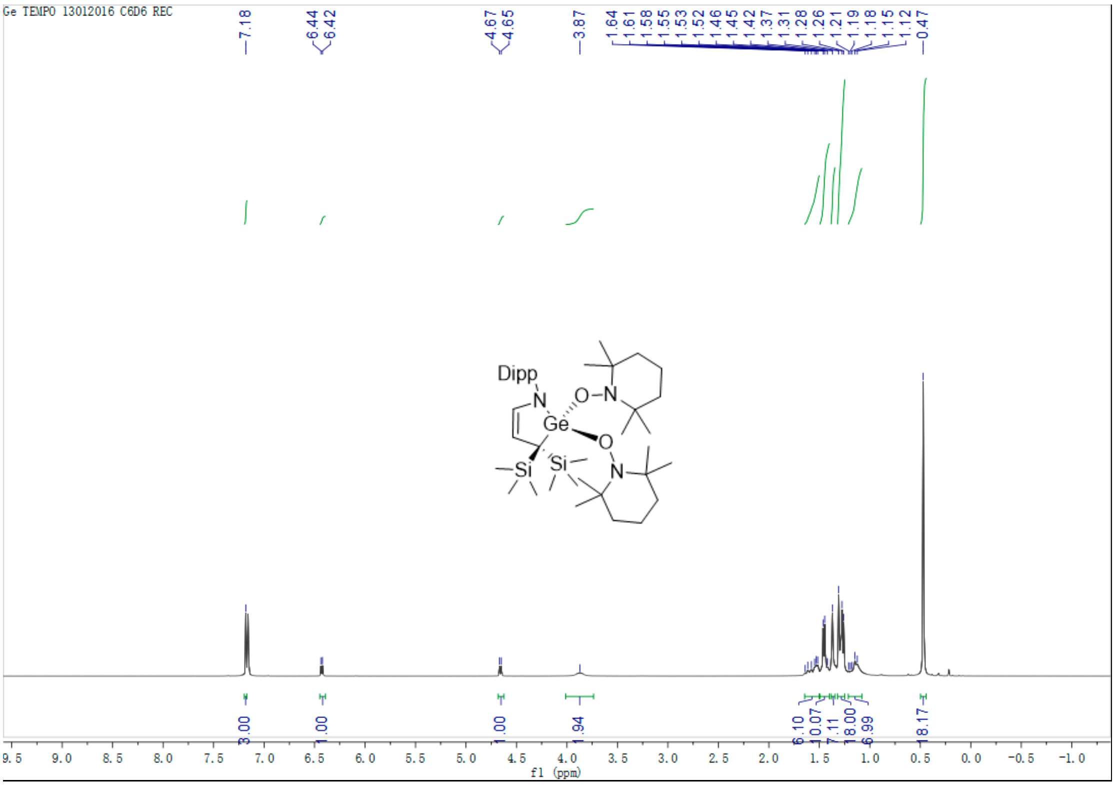

Figure S9. ^{1}H-NMR spectrum of **4.**

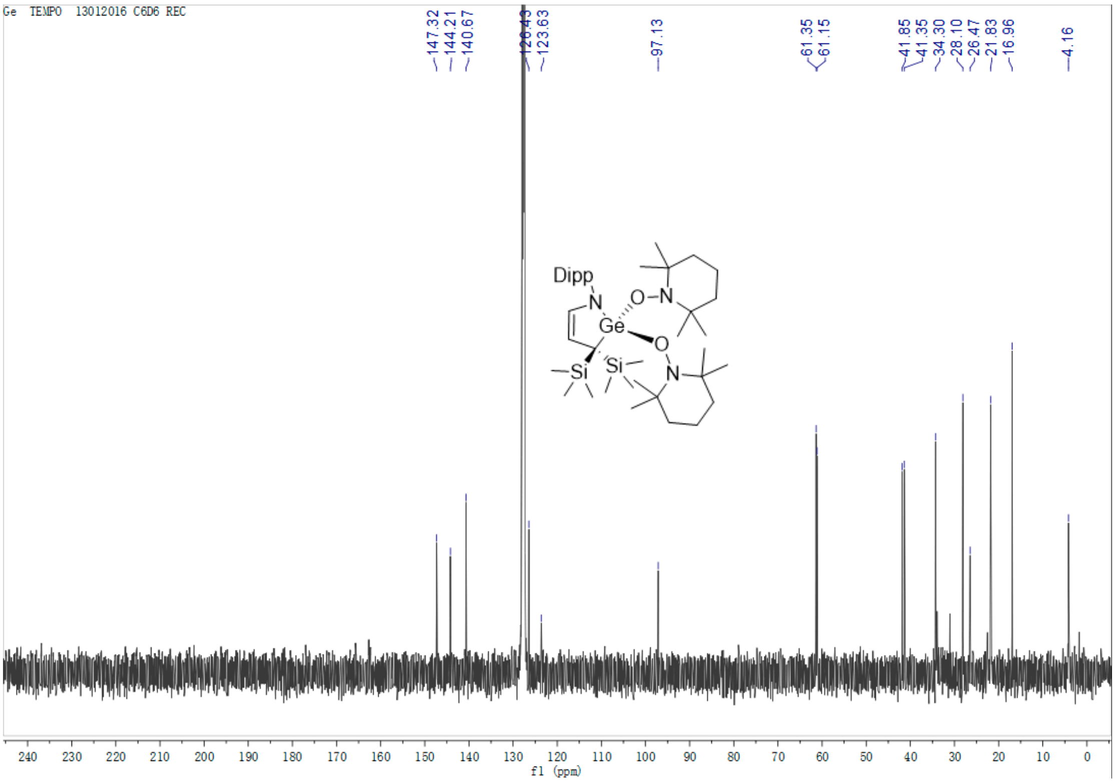

Figure S10. ^{13}C-NMR spectrum of **4.**

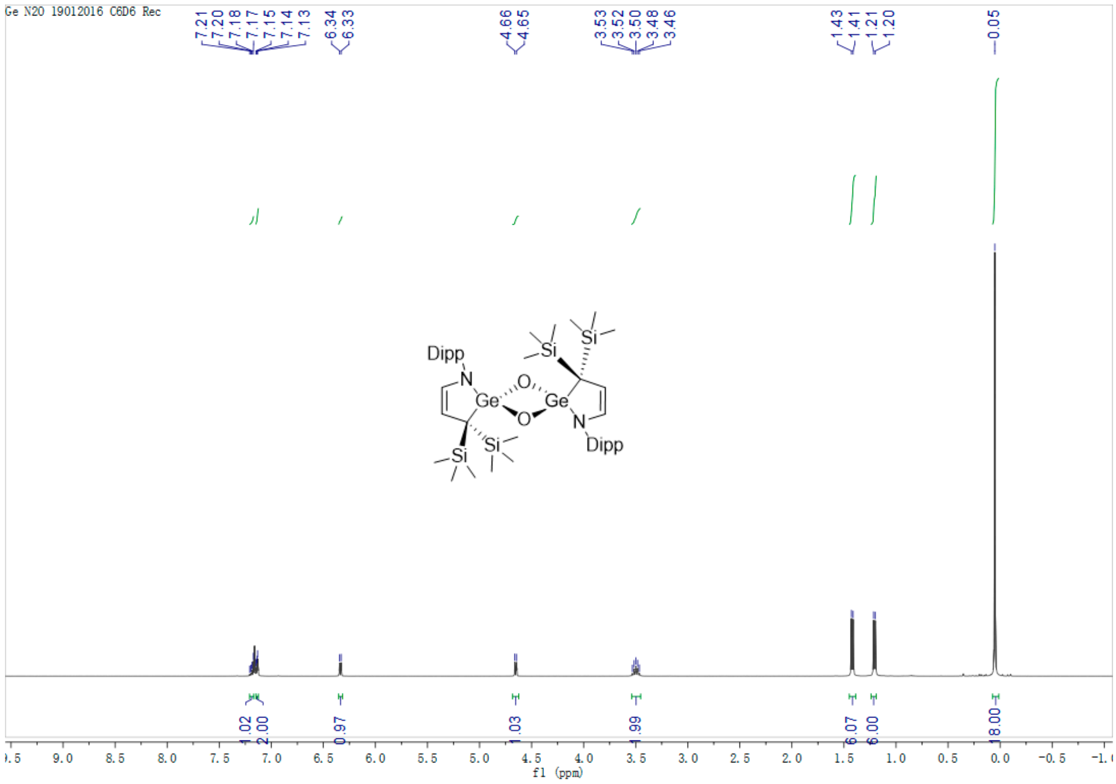

Figure S11. ^{1}H-NMR spectrum of **5.**

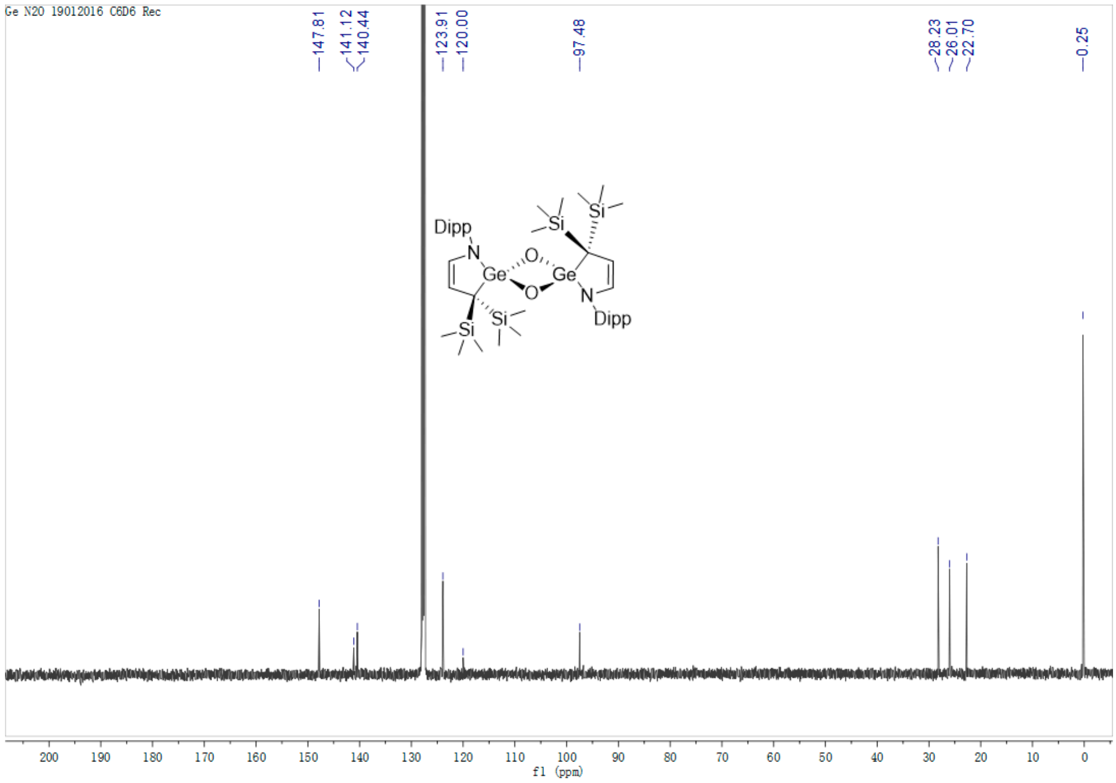

Figure S12. ^{13}C-NMR spectrum of **5.**

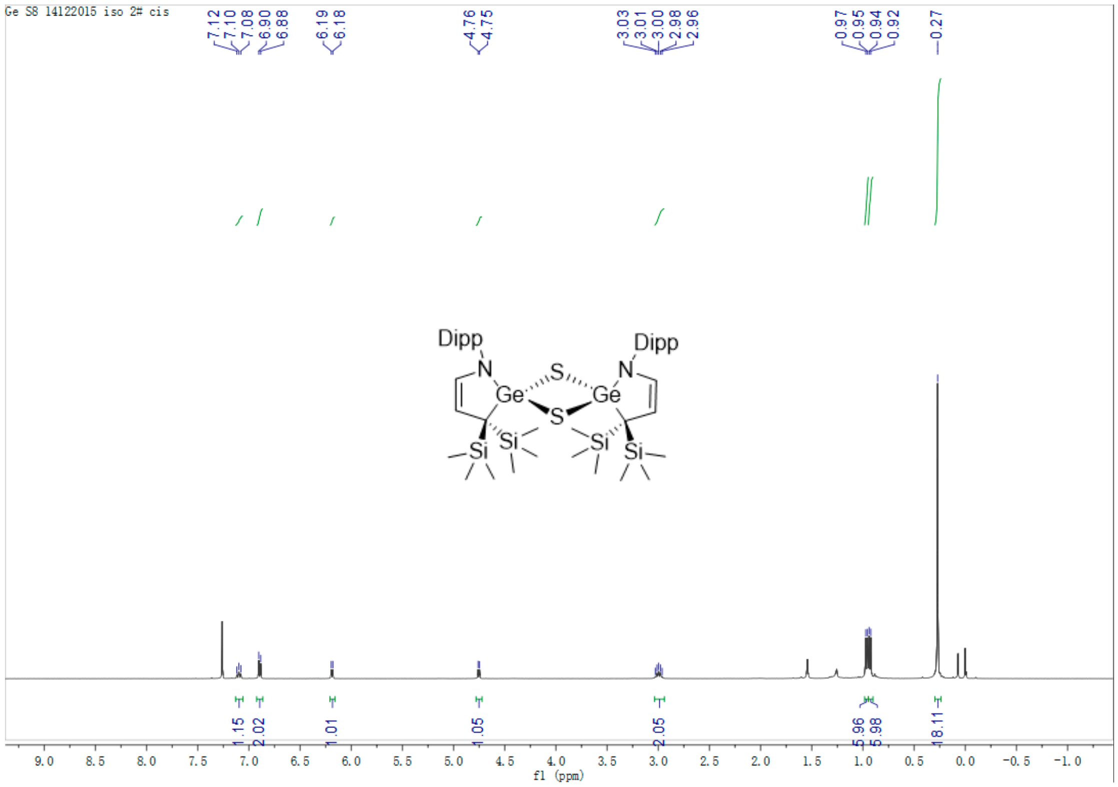

Figure S13. ^{1}H-NMR spectrum of **6a.**

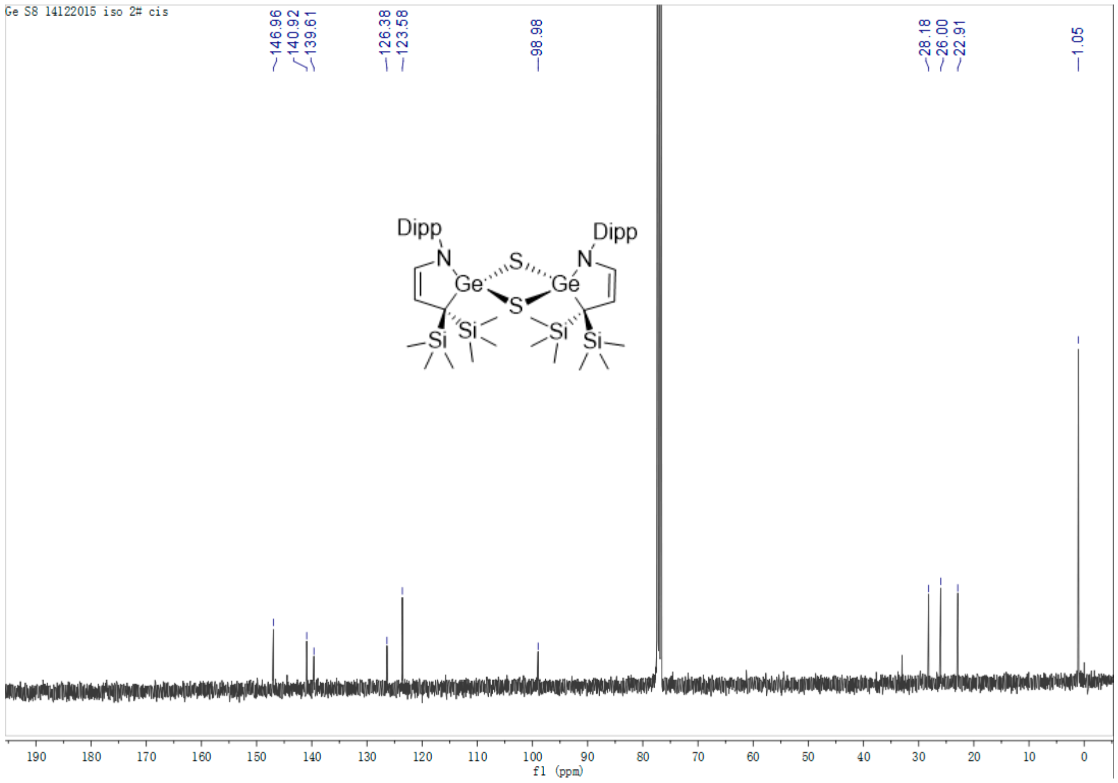

Figure S14. ^{13}C-NMR spectrum of **6a.**

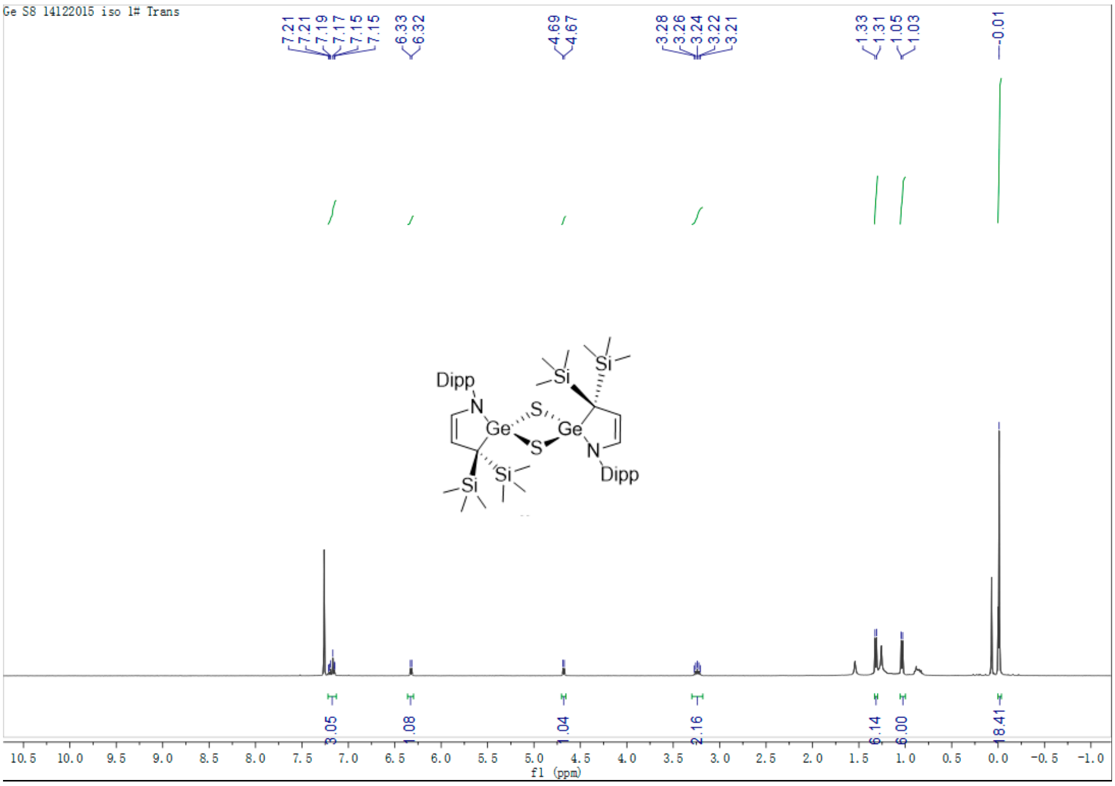

Figure S15. ^{1}H-NMR spectrum of **6b.**

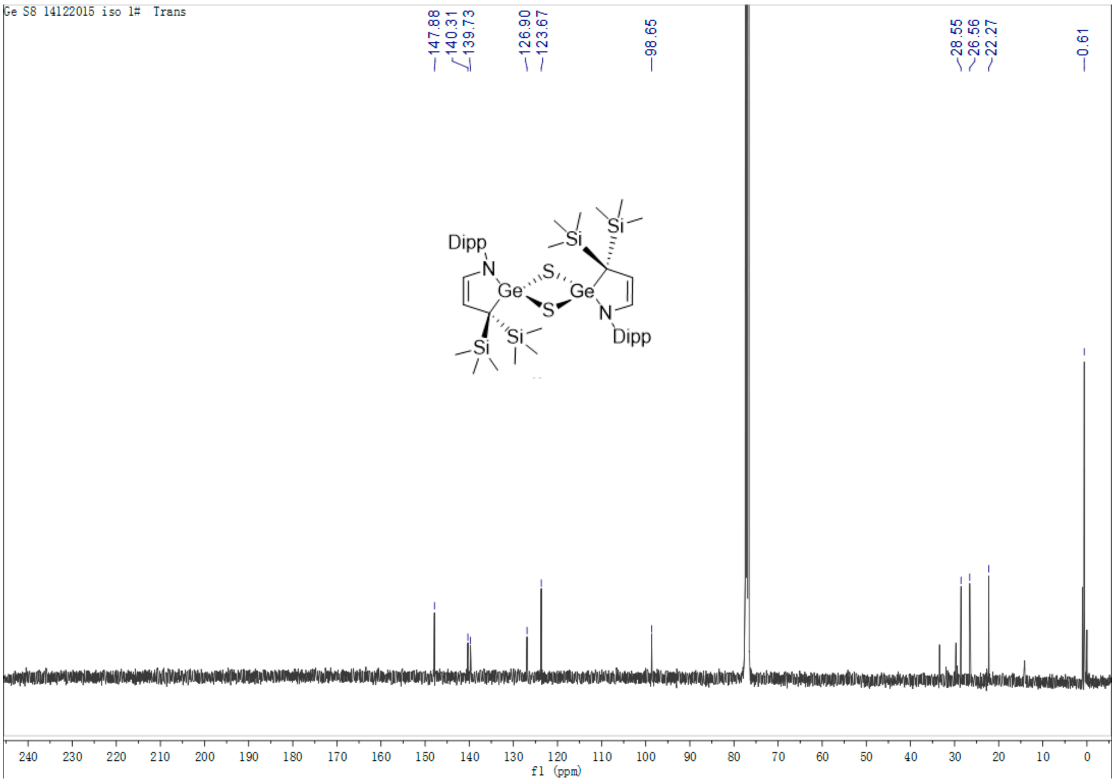

Figure S16. ^{13}C-NMR spectrum of **6b**.

3. UV-Visible Spectroscopy

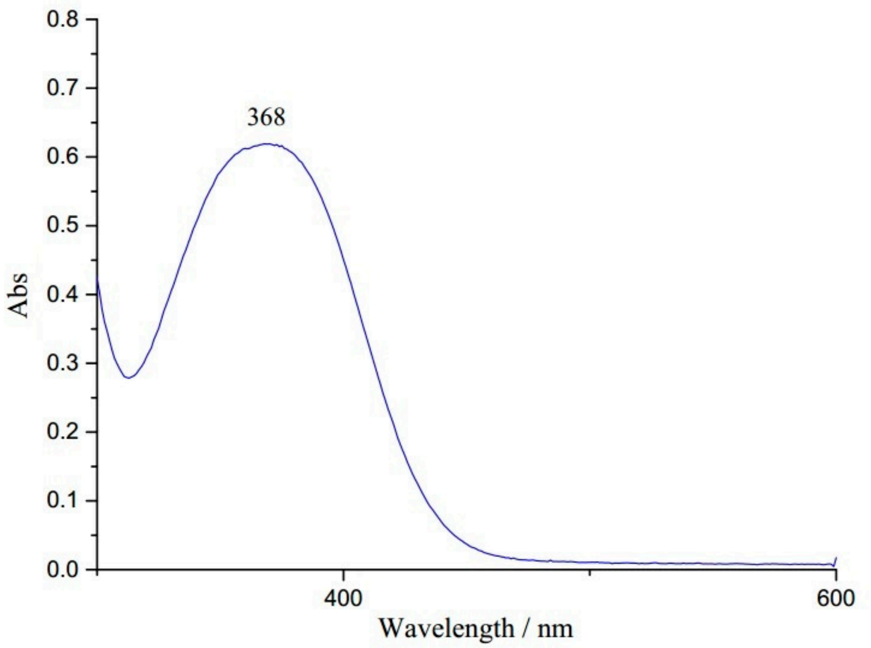

Figure S17. UV-visible spectrum of compound **3** in Hexane.

4. Theoretical Calculation

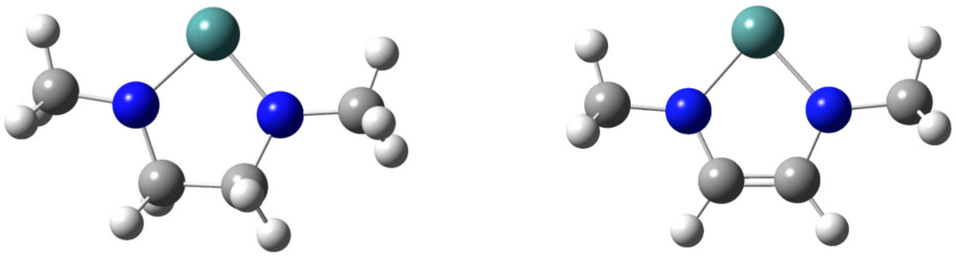

A **B**

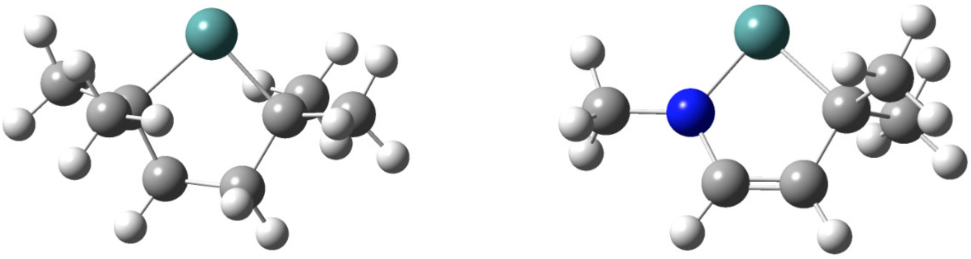

C **D**

A

C	0.74302498	−1.61271022	0.18178240
N	1.27078519	−0.28435381	−0.11946238
Ge	0.00000048	1.05725558	−0.00000009
N	−1.27078570	−0.28435276	0.11946059
C	−0.74302572	−1.61270976	−0.18178233
C	2.70556464	−0.12887246	−0.01946194
C	−2.70556527	−0.12887186	0.01946352
H	1.27987150	−2.39347663	−0.37575975
H	0.86702911	−1.83483303	1.25662167
H	−1.27987376	−2.39347566	0.37575903
H	−0.86702818	−1.83483292	−1.25662180
H	3.22979845	−0.75885342	−0.75335152
H	2.98843889	0.91086252	−0.21056523
H	3.08799469	−0.39982450	0.97864251
H	−2.98843790	0.91086652	0.21055087
H	−3.08799908	−0.39983931	−0.97863532
H	−3.22979727	−0.75884052	0.75336505

B

C	0.68102740	1.61109497	−0.00016495
N	1.25624747	0.36023206	−0.00016839
Ge	0.00000878	−1.04180876	−0.00008846
N	−1.25624751	0.36020679	−0.00007150
C	−0.68106474	1.61107484	−0.00013608
C	2.70532766	0.23370681	0.00035432
C	−2.70532733	0.23368961	0.00031830
H	1.29984116	2.50140102	−0.00021973
H	−1.29989169	2.50137188	−0.00015850
H	2.98404762	−0.82322362	−0.00057917
H	3.14823116	0.70115377	−0.88806874
H	3.14746703	0.69941176	0.89008155
H	−3.14811930	0.70110963	−0.88817825
H	−2.98406200	−0.82323772	−0.00057224
H	−3.14757252	0.69942433	0.88997542

C

C	−0.69800882	1.63386532	0.31890426
C	0.69802275	1.63385319	−0.31897117
C	1.41225412	0.30289706	−0.02017543
Ge	0.00000674	−1.12791531	−0.00000954
C	−1.41224700	0.30289572	0.02016918
C	−2.62593162	0.06982692	0.91994103
C	−1.80965946	0.20153355	−1.47305365
C	2.62598909	0.06981866	−0.91987507
C	1.80956603	0.20156065	1.47307930
H	−1.29005961	2.49814313	−0.02077098
H	−0.59946038	1.74465552	1.40694352
H	1.29007419	2.49814427	0.02066813
H	0.59947466	1.74458792	−1.40701627
H	−3.09532402	−0.90040683	0.72169637
H	−2.33863738	0.07736782	1.97787721
H	−3.39454153	0.84458672	0.78113572
H	−2.41058004	−0.69377080	−1.66653019
H	−2.39614922	1.07675527	−1.78637966
H	−0.94508493	0.15055867	−2.15471931
H	3.09534806	−0.90042715	−0.72161350
H	2.33876198	0.07737966	−1.97782973
H	3.39460828	0.84455937	−0.78101201
H	2.41048553	−0.69373065	1.66661218
H	2.39601307	1.07679809	1.78644043
H	0.94494514	0.15058226	2.15469254

D

C	−0.80381010	1.61838581	−0.00010535
C	0.53673360	1.64579561	−0.00015341
C	1.24333285	0.31672105	−0.00004152
Ge	−0.23610794	−1.06506190	−0.00011557
N	−1.43092639	0.35870233	−0.00004714
C	−2.88478947	0.31609906	0.00004610
C	2.10344760	0.13757283	−1.26887990
C	2.10254083	0.13747129	1.26945438
H	−1.44243390	2.49936367	−0.00009278
H	1.08756214	2.58444311	−0.00023321
H	−3.22262137	−0.72280441	0.00016020
H	−3.30040230	0.81006375	0.88838380
H	−3.30050696	0.80990438	−0.88833225
H	2.57739752	−0.85079620	−1.29773738
H	1.50328234	0.24072528	−2.18041061
H	2.90383540	0.88916136	−1.31423948
H	2.57642179	−0.85092301	1.29862806
H	2.90293453	0.88901527	1.31541261
H	1.50173765	0.24063751	2.18056760

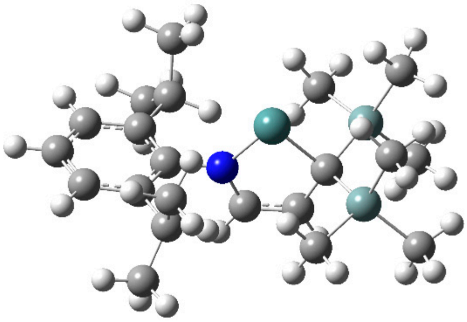

3

C	−2.28607731	0.00346427	0.19962120
C	−2.98328133	−1.22148539	0.10827528
C	−2.96603352	1.23958262	0.12632849
N	−0.85541345	−0.00640760	0.33178060
C	−4.35658094	1.22395475	−0.03289927
C	−2.21290262	2.56388012	0.17317321
C	−2.80317342	3.55232621	1.19313361
C	−2.13014195	3.19512977	−1.23114293
C	−5.05877948	0.02539167	−0.11805285
C	−4.37365140	−1.18393390	−0.05012634
C	−2.24978650	−2.55711213	0.13505467
C	−2.19140991	−3.17582164	−1.27579053
C	−2.84434678	−3.54543821	1.15272837
C	1.80288122	−0.00279849	0.32935939
C	1.08504393	−0.00374209	1.64076734
Si	2.76224173	−1.64935188	0.11986794
Si	2.77097272	1.63536056	0.08752416
Ge	0.39422639	−0.00428565	−1.07221312
C	3.46364194	1.80754428	−1.67107080
C	1.58432386	3.09032847	0.35002145
C	4.19814375	1.80789966	1.32527102
C	3.64554577	−1.74222783	−1.55614496
C	1.54129356	−3.10011879	0.18201470
C	4.01968109	−1.90848226	1.51415671
C	−0.26665204	−0.00710077	1.59473897
H	−1.19058149	2.34026896	0.48310470
H	−3.80984720	3.87994599	0.91218134

H	−2.17560301	4.44759793	1.25868898
H	−2.86380621	3.10699702	2.19114060
H	−3.12718355	3.41422864	−1.62897173
H	−1.63045852	2.52060918	−1.93457212
H	−1.56528908	4.13371902	−1.19989578
H	−4.89581884	2.16450309	−0.09630865
H	−6.13802439	0.03395348	−0.24175515
H	−4.92588313	−2.11592622	−0.12671061
H	−1.22091652	−2.35148325	0.43557517
H	−2.88517875	−3.10887402	2.15561465
H	−2.23177475	−4.45212413	1.20201820
H	−3.85997806	−3.85268189	0.88108410
H	−3.19561802	−3.38172321	−1.66253854
H	−1.63471333	−4.11956345	−1.26093398
H	−1.69501634	−2.49850621	−1.97884311
H	3.86605748	2.81847410	−1.80521080
H	2.68384935	1.65808992	−2.42616216
H	4.27160753	1.10297913	−1.88457264
H	1.02427174	2.99749645	1.28490862
H	0.86549194	3.16300043	−0.47294106
H	2.13992076	4.03401945	0.38743719
H	4.60115449	2.82669169	1.28651131
H	5.02134503	1.12150128	1.10359670
H	3.87526882	1.62024338	2.35455347
H	4.52490890	−1.09404505	−1.60985568
H	2.97147173	−1.46202089	−2.37328538
H	3.98361477	−2.76853100	−1.73952743
H	2.08725971	−4.04982984	0.21849067
H	0.89981399	−3.12391385	−0.70542030
H	0.90042382	−3.05499488	1.06745497
H	4.81531469	−1.15797077	1.50754668
H	4.48985249	−2.89324038	1.40917218
H	3.53997578	−1.87740750	2.49806782
H	−0.91968901	−0.00818133	2.46342179
H	1.60499705	−0.00287069	2.59530205

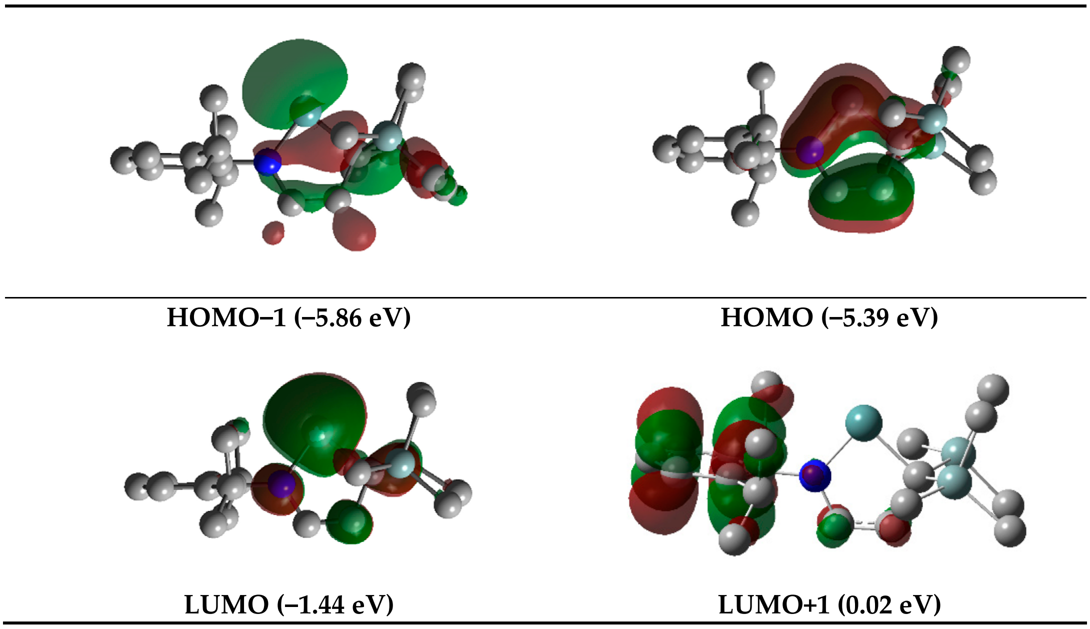

Figure S18. Plots of the frontier orbitals of compound **3** (hydrogen atoms are omitted for clarify). Orbital energies are shown in parentheses.

Table S2. NPA charges of **3**.

			Natural Population			
**Atom**	**No**	**Natural Charge**	**Core**	**Valence**	**Rydberg**	**Total**
C	1	0.14991	1.99884	3.83234	0.01892	5.85009
C	2	−0.01946	1.99898	4.00393	0.01655	6.01946
C	3	−0.01584	1.99901	4.00056	0.01627	6.01584
N	4	−0.80335	1.99948	5.78784	0.01603	7.80335
C	5	−0.21607	1.99916	4.20545	0.01146	6.21607
C	6	−0.23359	1.99922	4.22307	0.01130	6.23359
C	7	−0.63561	1.99944	4.62825	0.00793	6.63561
C	8	−0.62464	1.99941	4.61753	0.00770	6.62464
C	9	−0.18825	1.99927	4.17640	0.01258	6.18825
C	10	−0.21396	1.99916	4.20331	0.01150	6.21396
C	11	−0.23009	1.99922	4.21932	0.01155	6.23009
C	12	−0.63009	1.99941	4.62292	0.00776	6.63009
C	13	−0.63224	1.99944	4.62483	0.00798	6.63224
C	14	−1.51370	1.99933	5.50057	0.01380	7.51370
C	15	−0.25800	1.99925	4.23820	0.02054	6.25800
Si	16	1.83901	9.99797	2.12702	0.03600	12.16099
Si	17	1.84585	9.99807	2.12015	0.03593	12.15415
Ge	18	0.93639	27.99327	3.05612	0.01422	31.06361
C	19	−1.14738	1.99942	5.13543	0.01254	7.14738
C	20	−1.16093	1.99943	5.14916	0.01235	7.16093
C	21	−1.16818	1.99943	5.15684	0.01191	7.16818
C	22	−1.14159	1.99941	5.12969	0.01249	7.14159
C	23	−1.16134	1.99942	5.14957	0.01234	7.16134
C	24	−1.15168	1.99942	5.13983	0.01244	7.15168
C	25	0.00919	1.99914	3.97491	0.01677	5.99081
H	26	0.23321	0.00000	0.76349	0.00330	0.76679
H	27	0.21074	0.00000	0.78759	0.00167	0.78926
H	28	0.22157	0.00000	0.77697	0.00146	0.77843
H	29	0.22075	0.00000	0.77763	0.00162	0.77925
H	30	0.21428	0.00000	0.78402	0.00170	0.78572
H	31	0.22057	0.00000	0.77792	0.00150	0.77943
H	32	0.21505	0.00000	0.78335	0.00160	0.78495
H	33	0.20517	0.00000	0.79285	0.00199	0.79483
H	34	0.20558	0.00000	0.79270	0.00172	0.79442
H	35	0.20531	0.00000	0.79271	0.00197	0.79469
H	36	0.23152	0.00000	0.76499	0.00349	0.76848
H	37	0.21989	0.00000	0.77858	0.00153	0.78011
H	38	0.21982	0.00000	0.77870	0.00148	0.78018
H	39	0.20889	0.00000	0.78945	0.00166	0.79111
H	40	0.21400	0.00000	0.78417	0.00182	0.78600
H	41	0.21626	0.00000	0.78206	0.00168	0.78374
H	42	0.22230	0.00000	0.77614	0.00156	0.77770
H	43	0.23877	0.00000	0.75997	0.00126	0.76123
H	44	0.23929	0.00000	0.75926	0.00145	0.76071
H	45	0.23324	0.00000	0.76542	0.00134	0.76676
H	46	0.23933	0.00000	0.75899	0.00168	0.76067
H	47	0.23685	0.00000	0.76168	0.00147	0.76315
H	48	0.23994	0.00000	0.75880	0.00125	0.76006
H	49	0.24221	0.00000	0.75644	0.00135	0.75779
H	50	0.23394	0.00000	0.76469	0.00137	0.76606
H	51	0.23849	0.00000	0.76011	0.00140	0.76151
H	52	0.23227	0.00000	0.76640	0.00133	0.76773
H	53	0.23971	0.00000	0.75886	0.00143	0.76029
H	54	0.23843	0.00000	0.76036	0.00121	0.76157
H	55	0.23906	0.00000	0.75970	0.00124	0.76094
H	56	0.23564	0.00000	0.76290	0.00147	0.76436
H	57	0.24121	0.00000	0.75708	0.00172	0.75879
H	58	0.23211	0.00000	0.76642	0.00147	0.76789
H	59	0.23899	0.00000	0.75971	0.00130	0.76101
H	60	0.23624	0.00000	0.76237	0.00138	0.76376
H	61	0.20092	0.00000	0.79769	0.00139	0.79908
H	62	0.20415	0.00000	0.79360	0.00225	0.79585
* Total *		0.00000	91.97358	137.59707	0.42935	230.00000
